# Grand Challenges in Nano-Based Drug Delivery

**DOI:** 10.3389/fmedt.2019.00001

**Published:** 2019-12-03

**Authors:** Gianfranco Pasut

**Affiliations:** Department of Pharmaceutical and Pharmacological Sciences, University of Padua, Padua, Italy

**Keywords:** nanomedicines, nanotechnology, controlled release, drug targeting, drug delivery, nanobiotechnology

As its applications continuously expand in many areas of life, nanotechnology has been receiving increasing attention from all directions. Applications of nanotechnology are so diverse and numerous that sometimes it may be difficult to stay updated with their developments. Just to mention a few: in the agriculture industry, there has been talk about new pesticides, targeted genetic engineering, agrochemical delivery, sensors to monitor soil; in the nutrition industry, we are hearing about nutraceuticals, nutrient delivery, mineral and vitamin fortification, drinking water purification; as far as the processing industry is concerned, much has been said about nanoencapsulation of flavors/aromas, gelation and viscosifying agents, nanoemulsions, anti-caking, sanitation of equipment and we could go on and on….

The most basic definition of nanotechnology is: the engineering of functional systems at the molecular level. Undoubtedly one of the most well-known and perhaps relevant application for nanotechnology is nanomedicine. The layman's definition of nanomedicine is quite simply: the application of nanotechnology to diagnose or treat diseases or to repair damaged tissue. Given their complexity and its implications for public health and human life, I think it is important to take a moment to examine how the experts define these terms. The European Medicine Agency (EMA) defined ***Nanotechnology*** as “the production and application of structures, devices and systems by controlling the shape and size of materials at nanometre scale. The nanometre scale ranges from the atomic level at around 0.2 nm (2 Å) up to around 100 nm.” It then went on to define ***Nanomedicine*** “*as the application of nanotechnology in view of making a medical diagnosis or treating or preventing diseases. It exploits the improved and often novel physical, chemical and biological properties of materials at nanometre scale*” (1).

In its guidance document “Considering Whether an FDA-Regulated Product Involves the Application of Nanotechnology” the Food and Drug Administration (FDA) specified that ““*products that involve the application of nanotechnology” or “nanotechnology products” mean products that contain or are manufactured using materials in the nanoscale range, as well as products that contain or are manufactured using certain materials that otherwise exhibit related dimension-dependent properties or phenomena*”.

According to the definition of the European Commission (EC), which was published in a Recommendation (2011/696/EU): “‘*Nanomaterial' means a natural, incidental or manufactured material containing particles, in an unbound state or as an aggregate or as an agglomerate and where, for 50 % or more of the particles in the number size distribution, one or more external dimensions is in the size range 1 nm*−*100 nm. In specific cases and where warranted by concerns for the environment, health, safety or competitiveness the number size distribution threshold of 50 % may be replaced by a threshold between 1 and 50 %*.” The Recommendation also goes on to specify that there are some derogations (for fullerenes, grapheme flakes and single wall carbon nanotubes), defines terms such as particle, agglomerate and aggregate, and provides information on how to determine the compliance with the definition on the basis of the specific surface area by volume. The Joint Research Centre of the European Commission (JRC), moreover, published three scientific and technical reports to clarify the Recommendation and to define the term “nanomaterial” [Rauscher et al. (2); Rauscher et al. (3); Roebben et al. (4)].

Nanomedicines have evolved into a variety of forms including, for example, nanoparticles, liposomes, micelles, nanoemulsions, polymer-protein/drug conjugates, dendrimers, nanocrystals, polymeric vesicles, antibody-drug conjugates, nanogels, nanotubes, some already approved for diagnostic and/or therapeutic use (5). Since Doxil, a PEGylated liposome delivering doxorubicin, was first approved by the FDA in 1995, nanomedicine research has been taking giant steps forward although the pathway to success has not been entirely straightforward, and even today some may argue that it has not fully lived up to its original promise to revolutionize therapy and diagnosis.

It is undeniable that nanomedicines present extraordinary advantages with respect to conventional medicines including those of offering superior efficacy, bioavailability, improved transport across the biological barriers and disease-targeting, better adsorption, prolonged circulation and blood concentration, reduced toxicity and immunogenicity, etc. (6). These features, which depend on their basic physicochemical properties, namely their size, surface/volume ratio, softness/hardness, and their surface characteristics, have been ameliorating the lives of many patients (7, 8). One of the requirements for progress in this field is, nevertheless, a thorough physicochemical characterization of the nanomedicine before *an vivo* evaluation is carried out. When research is performed using proper standards and correct controls it accumulates precious knowledge for the entire scientific community. While the guidance of regulatory agencies is important for identifying the information needed for new drug applications (NDAs), the diversity and complexity of the different nanomedicine approaches have been obstructing the realization of a general guidance protocol. In fact, FDA addresses this point with dedicated industry documents such as the “*Liposome Drug Products*,” which provides information about chemistry, manufacturing, and controls, human pharmacokinetics, bioavailability, and labeling documentation (9), and “*Drug Products, Including Biological Products, that Contain Nanomaterials*” (10). Further documents are expected to provide vital information for the development of new nanomedicine approaches. A recent paper claims “*the variability of published literature with regards to characterizations performed and experimental details reported*” as one of the cause of the difficulties in the progress of nanotechnology for therapeutic applications (11). We need to harmonize the characterization of nanomedicines by performing standard protocols and by properly reporting the details of the experiments, thus promoting comparability between different approaches and reproducibility of the data.

If we examine the majority of the nanomedicines that have been studied until now we realize that for the most part they are nanoformulations of already approved drugs. It is important to remember that nanomaterials possess specific physiochemical properties due to their small size leading not only to the advantages listed above but also to concerns about safety and toxicity. In fact, since nanosystems interact in different ways to biological components it is vital to test for toxicity using validated methods tailored to the nanomaterials (12). As explained above, the lack of documents as guidance for industry could deter future developments of nanomedicines and delay the translation from the bench-to-bedside (13, 14). It could also explain why researchers have often concentrated on nanomedicine approaches that have already been approved as it is a way to stay on the safe side and to avoid unexpected pitfalls (14). Decision makers at all levels including the political one are called upon to support innovative research and to sustain the development of truly innovative nanomedicines. On their part, scientists should strive to investigate new and original products using a multidisciplinary approach to evaluate their research.

Although a large proportion of nanomedicines has been dedicated to cancer therapy and diagnosis given the ever rising number of that patient population, nanotechnology could constitute a valuable resource for innumerable other diseases, and the new applications that are developed can help to advance research horizons. Getting back to cancer therapy, which is the application of nanomedicine that most people are familiar with, nanotechnology has created delivery systems for selectively targeting cancer cell. Here is one of nanomedicines greatest challenges: efficaciously and safely delivering drugs to cancer cells.

Until now most of the approved and investigated nanomedicines have been unable to completely fulfill the promise of selective targeting to diseased tissues or cells, as analyzed in details for nanoparticles in the review of Wilhelm et al. (15). This review describes how the promises of better selective therapy with nanoparticles have not yet been accomplished owing the several difficulties for their delivery into tumors. Although the points discussed in this review are very important for a better design of particulate drug delivery systems, we might remember that the advantages of a nanomedicine approach is also going beyond the targeting. It can, for example, reduce the toxic side effects of the drug, reduce its degradation, permit it administration in water solution, etc. Then, beside the efforts directed in increasing the percentage of nanomedicine injected dose accumulating into the tumors, it would be relevant to study and promote the residence time of the drug in the tumors. The nanomedicines can work in this direction too. Nevertheless, the ambition of a selective disease delivery is very important and the path toward this goal is still long, as it has been for the development of antibody-drug conjugates that collected several failures before becoming a great therapeutic opportunity.

Targeting may be achieved exploiting nanomedicines' intrinsic features, such as size, charge, or through a specific recognition of a moiety composing the nanomedicine. Usually, targeting is carried out most effectively via direct functionalization of the nanomedicines' surface/backbone with a targeting agent/moiety.

As is well-known, targeting is usually classified into two types: passive targeting and ligand-mediated- (or active) targeting; nanomedicine can provide an extremely effective approach by exploiting both modality. Passive targeting to solid tumors and inflamed tissues can occur through anatomical and physiological changes in the tumor blood vessels together with the action of mediators regulating blood flow, and it is defined enhanced permeability and retention (EPR) effect (16). When ligand-mediated targeting is used, recognition takes place in a very specific manner on the surface of a nanomedicine with a targeting moiety interacting with its partner moiety (i.e., ligand-receptor or receptor-ligand interactions) to promote site-specific or cell-specific retention and accumulation. As explained above, nanomedicines are able to exploit both types of targeting modalities. For example, thanks to their size, they can improve biodistribution, pharmacokinetic and tumor extravasation achieving an accumulation in proximity to the cancer cells and, thanks to targeting moiety, they can interact with cancer cells of the tumor stroma resulting in a higher retention and internalization of drug release with the cancer cells (17).

EPR has been for many years the driving force for the development of many nanomedicines, especially nanoparticulate drug delivery systems. Currently, it is known that the positive advantages of the EPR effect might be affected by several parameters like circulating plasma concentration of the nanomedicine, its stability and plasma half-life, the tumor type, tumor region, the presence of intratumoral necrotic or inflamed area, and tumor vascularization (18, 19). Another important issue for EPR effect is the patient-to-patient variability, which depends on a patient's pathological and physiological characteristics and clinical condition (20–22). There is an intense debate about the real value of the EPR effect in patients. Some studies support the EPR-mediated accumulation of delivery systems within tumors, while others show that the EPR effect depends on the tumor model, suggesting that the EPR effect alone may not provide the entire solution.

The final point I would like to touch upon is the complexity and often the *over*-complexity of some nanomedicines. I frequently find myself reading research papers reporting on applications based on a combination of agents and targeting systems for theranostic effects that are stimuli responsive, guided by external stimuli, etc. Although these types of sophisticated applications may at first seem advantageous, as also already been highlighted by others, a more critical evaluation might uncover problems in the characterization or relevance of some of the components posing serious concerns about the real applicability of the system and its reproducibility (23). If, on the one hand, a certain degree of complexity is clearly necessary to efficaciously interact with biological systems and to fight against very multifaceted diseases, on the other, added features should be pursued with an eye for balancing the increased complexity with the risk of jeopardizing therapeutic outcome. Complexity should not be sought only for the sake of novelty (24).

Together with members of the journal's editorial board, I am looking forward to being a part of this section of “*Nano-based Drug Delivery*” under “*Frontiers in Medical Technology*” that will no doubt address many of the challenges facing nanomedicine research and pointing in the direction of future important developments in this field.

## Author Contributions

The author confirms being the sole contributor of this work and has approved it for publication.

### Conflict of Interest

The author declares that the research was conducted in the absence of any commercial or financial relationships that could be construed as a potential conflict of interest.
